# Structural insights into a unique preference for 3′ terminal guanine of mirtron in *Drosophila* TUTase tailor

**DOI:** 10.1093/nar/gky1116

**Published:** 2018-11-08

**Authors:** Lin Cheng, Fudong Li, Yiyang Jiang, Hailong Yu, Changlin Xie, Yunyu Shi, Qingguo Gong

**Affiliations:** 1 Hefei National Laboratory for Physical Science at the Microscale, School of Life Sciences, University of Science and Technology of China, 96 Jinzhai Road, Hefei, Anhui 230027, China; 2 High Magnet Field Laboratory, Chinese Academy of Science, 50 Shushanhu Road, Hefei, Anhui 230031, China

## Abstract

Terminal uridylyl transferase (TUTase) is one type of enzyme that modifies RNA molecules by facilitating the post-transcriptional addition of uridyl ribonucleotides to their 3′ ends. Recent researches have reported that *Drosophila* TUTase, Tailor, exhibits an intrinsic preference for RNA substrates ending in 3′G, distinguishing it from any other known TUTases. Through this unique feature, Tailor plays a crucial role as the repressor in the biogenesis pathway of splicing-derived mirtron pre-miRNAs. Here we describe crystal structures of core catalytic domain of Tailor and its complexes with RNA stretches 5′-AGU-3′ and 5′-AGUU-3′. We demonstrate that R327 and N347 are two key residues contributing cooperatively to Tailor's preference for 3′G, and R327 may play an extra role in facilitating the extension of polyuridylation chain. We also demonstrate that conformational stability of the exit of RNA-binding groove also contributes significantly to Tailor's activity. Overall, our work reveals useful insights to explain why *Drosophila* Tailor can preferentially select RNA substrates ending in 3′G and provides important values for further understanding the biological significances of biogenesis pathway of mirtron in flies.

## INTRODUCTION

Non-templated nucleotide addition to the 3′ end (or tailing) of RNA has been long recognized as an important and widespread type of RNA modification (1–3). Post-transcriptional polyadenylation has often been associated with 3′ ends of mRNAs and noncoding RNAs in different organisms of almost all species, triggering variable biological effects on the fate of the RNA molecules, such as maturation, translation, and degradation (4,5). Uridylation is probably the second popular theme in the events of RNA tailing while cytidylation and guanylation are much less reported in current researches (6,7). In the recent years, except numerous mRNAs (8–10), the physiological targets of uridylation have been progressively expanding to different noncoding RNAs including miRNAs, pre-miRNAs, gRNAs, piRNAs, snRNAs and small RNAs with alternative biological outcomes such as RNA editing, maturation and quality control (11–14). In spite of diverse RNA substrates and alternative functional consequences on which it impacts, uridylation seemingly has a prevailing role in mediating the process of RNA degradation (15).

Terminal uridylyltransferases (TUTases) catalyzing the non-templated addition of uridines have been identified in many eukaryotes. Accompanied by the function studies and atomic-level structure determinations of a series of TUTases from different species in the last decade (16–22), the catalytic mechanism of uridylation by TUTase has been progressively characterized. Similar to other terminal nucleotidyltransferases belonging to the DNA polymerase β superfamily, all TUTases contain two canonical domains, a Polβ nucleotidyltransferase domain at the N-terminal side and a poly(A) polymerase-associated domain (PAP) at the C-terminal side, to constitute the core catalytic region (23–25). Apart from the core region, most TUTases also have some non-catalytic domains, such as RNA-binding domains RRM and Zinc Finger (ZnF), and intrinsically disordered regions (IDRs), possibly reflecting a fast-evolving adaptability in their RNA substrate recognition (15,18,23,26).

Among the already known TUTases, Cid1 from *Schizosaccharomyces pombe* is a well-characterized one and has been shown to be crucial in promoting mRNA degradation (27–31). A dozen of crystal structures with different resolutions have been determined for Cid1 or its complexes with UTP in the last a few years to elucidate its catalytic mechanism (20–22,32,33). As the mammalian homologues to Cid1, TUT1, TUT4 (ZCCHC11), and TUT7 (ZCCHC6) have been shown to specifically regulate the stabilities of U6 snRNA and pre-let-7 miRNAs (28,34–37) besides their general role in catalyzing the oligouridylation of mRNAs (34,38,39).

Tailor is so far the only experimentally identified TUTase in flies and has been recently proven to be responsible for the majority of uridylation signatures in miRNAs and pre-miRNAs of S2 cells, especially mirtron RNAs (40,41). It forms a stable complex with the homologue protein of the human Perlman syndrome exoribonuclease Dis3l2 in *Drosophila* (dmDis3l2), functioning cooperatively in the 3′-to-5′ degradation of structural RNAs (42). However, one intriguing characteristic of Tailor is that it exhibits an intrinsic selectivity for RNA substrates ending in 3′G (40,41).

Mirtron is one group of non-canonical pre-miRNA-like hairpins which have been found in flies, worms, and humans (43). Mirtrons are produced via a Drosha-independent pathway primarily from the splicing of short introns and usually bear the conserved splice acceptor sequence AG at the 3′ end (44,45). In fact, mirtrons have been identified as the important portion of the newly-evolved miRNAs pool in both insects and vertebrates (46,47). Their low accumulation and high evolutionary variation compared to the canonical miRNAs (46) seem to be inconsistent with the conservation of the mirtron pathway found in diverse species, suggesting the existence of a mechanism antagonizing the biogenesis of this group of non-canonical small RNAs. The identification of Tailor and its function to preferentially suppress mirtron well resolved the above speculation. It has been further evidenced that, while non-conserved miRNAs show no sequence preferences for their 3′ end nucleotide, conserved miRNAs are notably deficient for 3′G and much less uridylated (40,41). All together, Tailor-mediated uridylation turns out to be a crucial mechanism in flies to impact on the evolution of both mirtron and canonical miRNAs.

All the crystal structures of TUTase catalytic domains determined so far indicated a conserved folding. Given that Tailor is a *Drosophila* TUTase with selectivity towards mirtron RNAs ending in 3′G, one remaining mystery that needs to be immediately revealed is what is the structural basis Tailor forms for this unique 3′G preference. In this research, we determine the high-resolution crystal structures of the core catalytic domain of *Drosophila* Tailor, and its complexes with two short RNA stretches 5′-AGU-3′ and 5′-AGUU-3′ which are designed to mimic the states of the first and second uridine addition at 3′G, respectively. Based on the careful analysis of protein-RNA interactions, we conclude that R327 and N347 are crucial residues contributing cooperatively to the intrinsic preference of Tailor for RNA substrates ending in 3′G, and R327 may play an extra role in facilitating the extension of polyuridylation chain. These results are further verified by the in-vitro nucleotide transferase assay. The unveiling of this unique mechanism in Tailor will definitely benefit the functional investigation of biogenesis pathway of mirtron in flies.

## MATERIALS AND METHODS

### Expression and purification

The residues 202–560 and 137–560 of Tailor (hereafter named Tailor-C and Tailor-ZnC) were respectively constructed to represent the core enzymatic domain alone (Supplementary Figure S1) and with a possible C2H2 Zinc finger domain at its N-terminus (48). Their nucleotide sequences were amplified by PCR from genomic DNA, cloned into pET28a for expression in *Escherichia coli* as the fusion proteins with N-terminal 8xHis-SUMO tag. The recombinant protein was overexpressed in *E. coli* Gold (DE3) cells, grown in LB media at 37°C to an OD_600_ of 0.8–1.0, induced at 16°C with 0.5 mM isopropyl-β-d-thiogalactopyranoside (IPTG). Cells were harvested by centrifugation after overnight, resuspended in buffer A (25 mM Tris–HCl, pH 7.0 and 1 M NaCl) containing 10 μg/ml DNase and 10 μg/ml RNase.

Cells were lysed by sonication and centrifuged at 16 000 × g for 30 min at 4°C. The supernatant was loaded onto a Ni-NTA column (QIAGEN) equilibrated with buffer A. Following a wash with Buffer B (25 mM Tris–HCl, pH 7.0, 1 M NaCl and 40 mM imidazole), the bound proteins were eluted in Buffer C (25 mM Tris–HCl, pH 7.0, 500 mM NaCl and 500 mM imidazole). The N-terminal 8× His-sumo tag was removed by ULP1 digestion in buffer D (25 mM Tris–HCl, pH 7.0, 200 mM NaCl and 2 mM β-mercaptoethanol) overnight at 8°C. Tailor-C or Tailor-ZnC was loaded directly onto a HiTrap Heparin HP column (GE healthcare) equilibrated with buffer D, eluted using a linear gradient of 10 column volumes to buffer A, and was further purified by size exclusion chromatography on a Superdex™ 200 column (GE healthcare) equilibrated with buffer A. The purified protein was then dialyzed against buffer E (25 mM Tris–HCl, pH 7.0, 150 mM NaCl, 2 mM DTT) and concentrated to 5 mg/ml. All mutations of Tailor-C were generated using PCR and MutantBEST kit (TaKaRa). The purification protocol for all mutants was same to the wild-type Tailor. Protease inhibitor Cocktail is added to prevent protein degradation in Tailor-ZnC after lysing cells.

### Crystallization and data collection

The crystals of Tailor-C were harvested in a sitting-drop vapor diffusion setup using a 2:1 or 3:1 ration of protein to reservoir at 4°C. The reservoir solution contains 0.1 M Tris–HCl, pH 8.4, 50 mM NaCl, 0.2 M lithium sulfate monohydrate and 15% PEG3350. The crystals of Tailor in complex with RNA stretches (5′-AGU-3′, 5′-AGUU-3′, and 5′-UUUU-3′) were obtained by soaking native crystals for 16 h with lithium chloride instead of lithium sulfate monohydrate in reservoir solution supplemented with 5 mM nucleic acids. The crystals of Tailor-C D343A mutant grew in a condition containing 0.1 M Tris–HCl, pH 8.4, 0.2 M NaCl, between 18% and 15% PEG3350 using a 2:1 ration of protein to reservoir at 4°C. The crystals of the complex of D343A with UTP were obtained by soaking crystals of D343A in crystallization buffer supplemented with 10 mM UTP and 20 mM MgCl_2_ for 10 min.

The crystals were cryo-protected by passing them into the respective reservoir solution supplemented with 35% (v/v) MPD before flash freezing in liquid nitrogen. Diffraction datasets for all crystals were collected on beamline 19U1 at Shanghai Synchrotron Radiation Facility (SSRF) at 100 K.

### Structure determination and refinement

X-ray intensity data of the crystals were processed with HKL2000 software (49), and initial phases for apo-form Tailor-C were obtained by molecular replacement using PhaserMR (50) and MOLREP (51) using the structure *sp*Cid1 (PDB ID: 4NKT) as the search model (32). The complex structures of Tailor with RNA stretches or UTP were determined by using the structure of apo-form Tailor as the search model. All models were built and refined using COOT (52) and Refmac5 (53) in the CCP4 package and Phenix (54). The qualities of the structures were validated using PROCHECK (55). All figures were prepared by PYMOL.

### In-vitro nucleotide transferase assay

**Figure 1.**
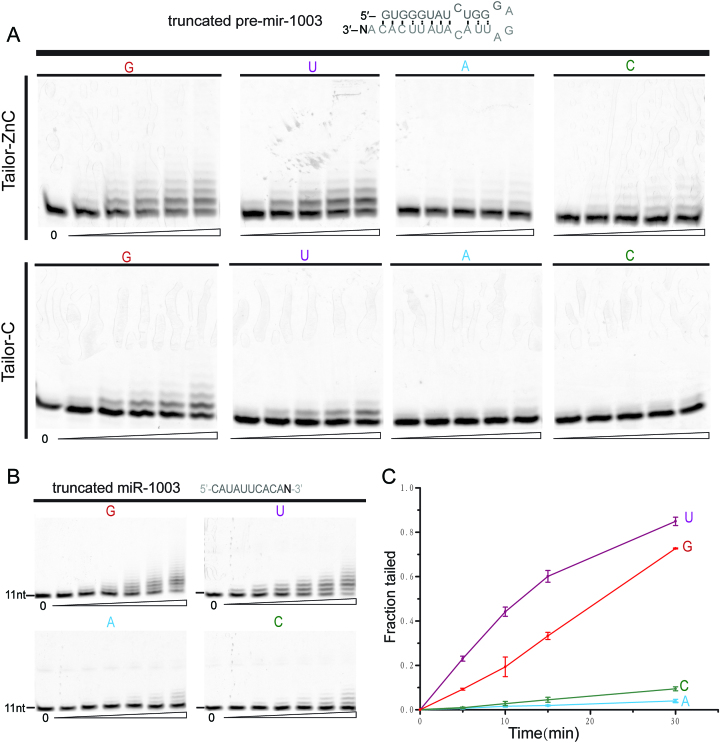
The core enzymatic domain of Tailor preferentially catalyzes the uridylation of mirtron RNAs ending in 3′G and 3′U. (**A**) *In-vitro* nucleotide transferase assay of truncated pre-mir-1003 RNAs bearing different 3′ nucleotides, by Tailor-C and Tailor-ZnC. The sequence of truncated mirtron pre-mir-1003 is displayed at the top of gel results. (**B**) *In-vitro* nucleotide transferase assay of truncated miR-1003 RNAs bearing different 3′ nucleotides. The sequence of truncated miR-1003 is displayed at the top of gel results. (**C**) Quantification of three independent replicates of the experiment shown in (**B**). Data presented as mean ± SD.

Purified Tailor-ZnC, Tailor-C and mutants were assayed for oligouridylation activity at 25°C in a buffer containing 25 mM Tris–HCl, pH 7.2, 100 mM NaCl, 10 mM MgCl_2_ and 5 mM DTT. 200 nM truncated miR-1003 (5′-Cy5-CAUAUUCACAG-3′), truncated pre-mir-1003 hairpin capped by a GAGA tetraloop (5′-Cy5-GUGGGUAUCUGGGAGAUUACAUAUUCACAG-3′), or their variants (3′ terminal G is replaced by A/C/U) with Cy5 probes labeled at the 5′ ends was mixed with 100 nM protein and 1mM UTP. The reaction was stopped at a series of time-points (1, 5, 10, 15, 30 min for pre-mir-1003; 1, 3, 5, 10, 15, 30 min for miR-1003) by the addition of EDTA (final concentration: 100 mM). Products were denatured by 95°C for 2 min and analyzed on 20% denaturing polyacrylamide gels run at 200 V for 2 h. RNA bands were imaged on a Typhoon FLA 7000 (GE Healthcare) by detecting Cy5, and quantified using ImageQuant TL (GE Healthcare). Rolling-ball algorithm was used for background subtraction. Datasets were processed with Originlab and the data for first two time-points (1 and 3 min) were not included in the Figure 1C due to larger errors.

### Electrophoretic mobility shift assay (EMSA)

EMSA were carried out in 20 μl reaction mixture containing 25 mM Tris–HCl, pH 7.2, 100 mM NaCl, 5 mM DTT, 500 nM truncated miR-1003 with Cy5 probe labeled at the 5′ end and the enzyme (Tailor-C and its mutants) at different concentrations (0, 0.156, 0.312, 0.625, 1.25, 2.5 μM). The reaction mixtures were incubated for 30 min on ice and then loaded on a 6% nondenaturing polyacrylamide gels, run at 4°C for 18 min at 120 V. Subsequently, the gels were imaged on a Typhoon FLA 7000 (GE Healthcare) by detecting Cy5.

### Circular dichroism (CD) spectroscopy

Far-UV spectra of Tailor-C and its mutants were recorded on an Applied Photophysics Chriascan spectrometer with the wavelength between 195 and 260 nm at 25°C. The concentrations of Tailor and its mutants were 0.1 mg/ml in 20 mM sodium phosphate buffer (pH 7.2) containing 100 mM NaCl and 5 mM DTT. A buffer-only sample was used as a reference and all the samples were tested in triplicate. Datasets were processed with Originlab. Our CD results confirmed that all mutants of Tailor-C used in this research are properly folded (Supplementary Figure S2)

## RESULTS

### Tailor preferentially catalyzes the uridylation of mirtron RNAs ending in 3′G and 3′U

Tailor has been recently identified as the enzyme responsible for the major events of uridylation in flies and proven to exhibit an intrinsic preference for pre-miRNA substrates ending in 3′G, especially mirtron hairpins (40,41). To confirm this result, we utilized an in-vitro nucleotide transferase assay to investigate the Tailor-induced uridylation of a truncated portion of pre-mir-1003 which is a classical mirtron previously identified as the RNA substrate for Tailor (40). Both truncated pre-mir-1003 bearing a terminal 3′G, and their three variants (ending in A/U/C) were subject to the tailing assay, respectively. As summarized in Figure 1A, our time course assays indicated that, although the tailing efficiencies of pre-mir-1003 and its variants by Tailor-ZnC are somewhat higher than those by Tailor-C, both of them share a similar catalytic tendency in which Tailor catalyzes most efficient tailing towards truncated pre-mir-1003 RNA bearing terminal G and less efficient tailing for that ending in U while the uridylation levels of those ending in A and C are much lower, perfectly consistent with the result of the previous research (40).

Although it has been proven that mirtron hairpins are preferred Tailor substrates, Tailor does catalyze the uridylation of their mature miRNAs both in vivo and in vitro (29,40–42). Therefore, the nucleotide transferase assay was also applied to the truncated miR-1003 and its variants in this research (Figure 1B and C). Our results showed that miR-1003 RNAs ending 3′G and 3′U exhibit much more efficient tailing by Tailor-C (72.7 ± 0.2% and 84.9 ± 1.9% tailed after 30 min) than their 3′A and 3′C variants (4.0 ± 0.7% and 9.4 ± 0.9% tailed after 30 min). Overall tailing profile of truncated miR-1003 RNAs by Tailor is similar to that of their double-stranded counterparts except the tailing efficiencies of miR-1003 and variants are all higher than those of pre-mir-1003 RNAs, inconsistent with the previous in vitro results by others that Tailor prefers substrate hairpins over mature miRNA (40,41). This discrepancy could be rationalized by the Cy5 probe we introduced at the 5′ end of pre-mir-1003 hairpin, which is very close to the 3′ terminal nucleotides and might affect their insertion into the active site of Tailor for uridylation. Another possibility is that the recombinant Tailor was used in our nucleotide transferase assay instead of the immunopurified Tailor used by others (40). Overall, our results of enzymatic assay indicated that the core catalytic domain of Tailor alone exhibits evident preference for the 3′G- and 3′U-ended RNA analogues to miR-1003 and its precursor.

### Overall structure of Tailor and its complexes with RNAs

**Figure 2.**
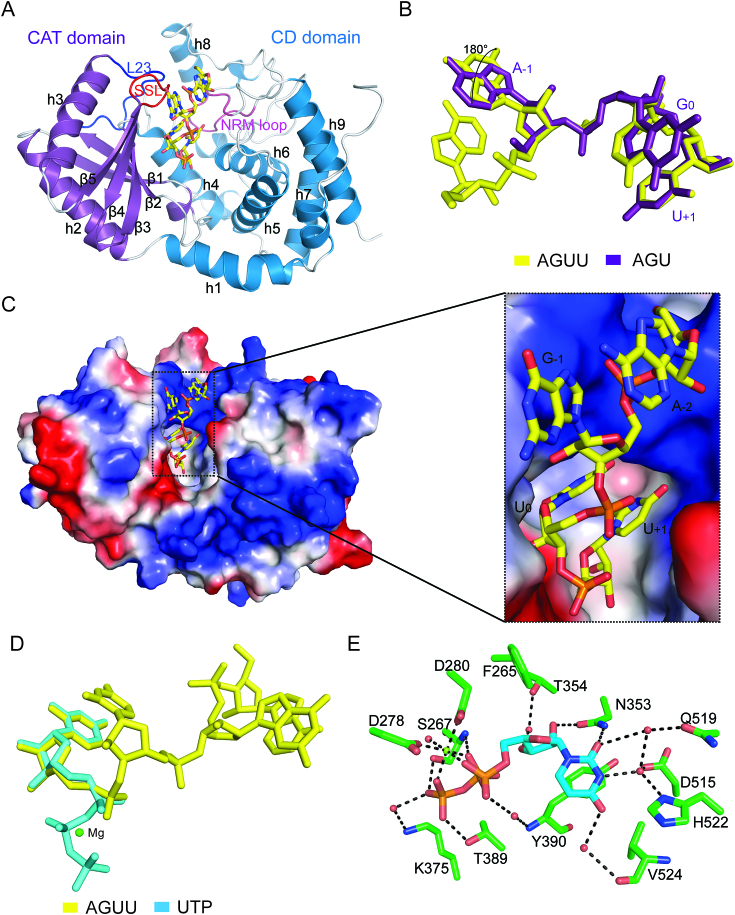
Crystal structures of Tailor-C and its complexes with UTP and RNA stretches 5′-AGU-3′ and 5′-AGUU-3′. (**A**) Cartoon representation of apo-form Tailor-AGUU. The juxtaposed CAT and CD domains are shown in purple and sky blue, respectively. Three important loops, including NRM loop for nucleotide recognition, SSL loop for substrate specificity, and L23 loop are shown in warm pink, red, and blue, respectively. (**B**) The structural superimposition of 5′-AGU-3′ and 5′-AGUU-3′. (**C**) The overview of 5′-AGUU-3′ lying along the catalytic groove of Tailor. Tailor-C is shown in its electrostatic surface potential, and RNA stretch is shown in stick model. (*Inset*) A close-up of the engagement of 5′-AGUU-3′ into the catalytic groove. (**D**) The structural superimposition of UTP and 5′-AGUU-3′. (**E**) The interaction details of UTP with surrounding residues in Tailor-UTP. UTP and Tailor residues are shown in cyan and green sticks, respectively. Hydrogen bonding interactions are all indicated as black dashed lines and water molecules are shown in red spheres.

In this research, we determined the high-resolution crystal structures of *Drosophila* Tailor-C, and its complexes with UTP and three short RNA stretches (5′-AGU-3′, 5′-AGUU-3′ and 5′-UUUU-3′) using X-ray crystallography (Table 1). The overall architectures of Tailor in all models are almost identical and resemble the previously reported structures of the other TUTases, belonging to noncanonical nuclear poly(A) polymerase of the DNA polymerase β superfamily. The structure of Tailor contains two canonical globular domains separated by a large catalytic groove, which serves to accommodate the 3′ ends of the target RNAs for the post-transcriptional addition of uridyl ribonucleotides (Figure 2A). The catalytic domain (CAT) of Tailor consists of a mixed five-stranded β-sheet (β1–β5 strands) packed tightly by two α-helices (h2 and h3) from one side, exhibiting a typical folding of the DNA polymerase β family enzyme. Three conserved catalytic aspartic acid residues (Asp278, Asp280 and Asp 343) sit closely with their side chains all pointing outward from the other side towards the groove. The central domain (CD) of Tailor, on the other hand, is made up of a long α-helix (h1) and a core region of six closely packed α-helices (h4–h9) which is a homologue to the central domain of PAP. The juxtaposed CAT and CD are physically connected through the linkers of h4–β5 and h1–h2. In addition, a highly conserved loop, which has been previously termed as nucleotide-recognition motif (NRM), also exists in the CD of Tailor.

**Table 1.** Data collection and refinement statistics

Data collection statistics	Tailor	Tailor-AGU	Tailor-AGUU	Tailor-U4	Tailor-UTP
**PDB ID**	5Z4C	5Z4A	5Z4D	5Z4J	5Z4M
**Data collection**					
2003 Wavelength (Å)	0.9778	0.9778	0.9778	0.9785	0.9785
Space group	*C222_1_*				
Cell dimensions					
*a, b, c* (Å)	62.02, 84.84, 148.67	61.72, 84.94, 148.26	61.57, 84.79, 148.12	61.63, 84.84, 148.31	61.96, 84.56, 149.35
Resolution range (Å)	50.00–1.65	50.00–1.64	50.00–1.80	50.00–1.82	50.00–1.74
	(1.68–1.65)*	(1.67–1.64)	(1.83–1.80)	(1.85–1.82)	(1.77–1.74)
*R*_merge_ (%)	6.0 (46.4)	7.5 (51.8)	8.0 (46.5)	8.3 (47.8)	7.1 (59.0)
*I/σI*	33.0 (3.5)	29.4 (4.3)	30.3 (3.3)	40.1 (6.80	38.8 (3.0)
Completeness (%)	99.4 (97.8)	100.0 (100.0)	98.9 (95.3)	99.6 (99.7)	99.7 (98.9)
Redundancy	12.0 (11.5)	13.1 (12.7)	12.5 (11.1)	13.1 (13.4)	12.9 (11.5)
**Refinement**					
No.reflections overall/test set	47238/2404	48203/2420	35673/1816	35158/1747	40347/1997
*Rwork/Rfree* (%)	16.9/19.7	15.7/19.1	15.8/18.4	15.5/19.2	16.7/20.6
Number of atoms					
Protein	5671	5776	5745	5646	5629
Ligands	0	96	126	119	81
Water	217	243	186	193	177
*B*-factors (Å^2^)					
Protein	35.95	32.42	34.28	39.74	46.65
Ligands	NA	31.16	43.18	55.09	68.81
Water	37.41	32.88	33.36	36.17	39.66
R.M.S. deviations					
Bond length (Å)	0.010	0.009	0.010	0.010	0.010
Bond angles (°)	0.952	0.979	1.009	1.029	1.043
Ramachandran plot					
Favored (%)	98.22	97.01	97.92	98.26	98.56
Allowed (%)	1.78	2.99	1.78	1.74	1.44
Outlier (%)	0.00	0.00	0.30	0.00	0.00

*Values in parentheses are for the highest resolution shell.

Three complex structures of Tailor-AGU, Tailor-AGUU and Tailor-U4 (Tailor-UUUU) we obtained in this research were respectively designed to mimic the post-catalytic states of the addition of the first and second uridines as well as the extension of polyuridylation chain. In all these structures, RNA stretches exhibit a very similar binding mode for Tailor (Figure 2B, C and Supplementary Figures S3 and S4), lying along the canonical catalytic groove formed between the CAT and CD of Tailor with the terminal 3′Us anchored into the pocket at the bottom of the groove which is usually occupied by UTP or its analogue in previously reported structures (19,21). This is further confirmed by our structure of Tailor-UTP in which UTP can be perfectly superimposed with the terminal 3′Us in Tailor-AGU and Tailor-AGUU (Figure 2D) and maintains conserved interactions with surrounding residues (Figure 2E). Moreover, the AGU of Tailor-AGU and the GUU of Tailor-AGUU show almost identical conformation for the sugar-phosphate backbone and very similar orientations for base planes between the corresponding nucleotides, except there is ∼180° relative rotation between the bases of adenine of AGU and guanine of AGUU about the glycosidic bond (Figure 2B). This extra adenine sits at the exit of RNA-binding groove of Tailor and shows no important interaction with the protein.

### Protein-RNA interaction details in Tailor-AGU and Tailor-AGUU

**Figure 3.**
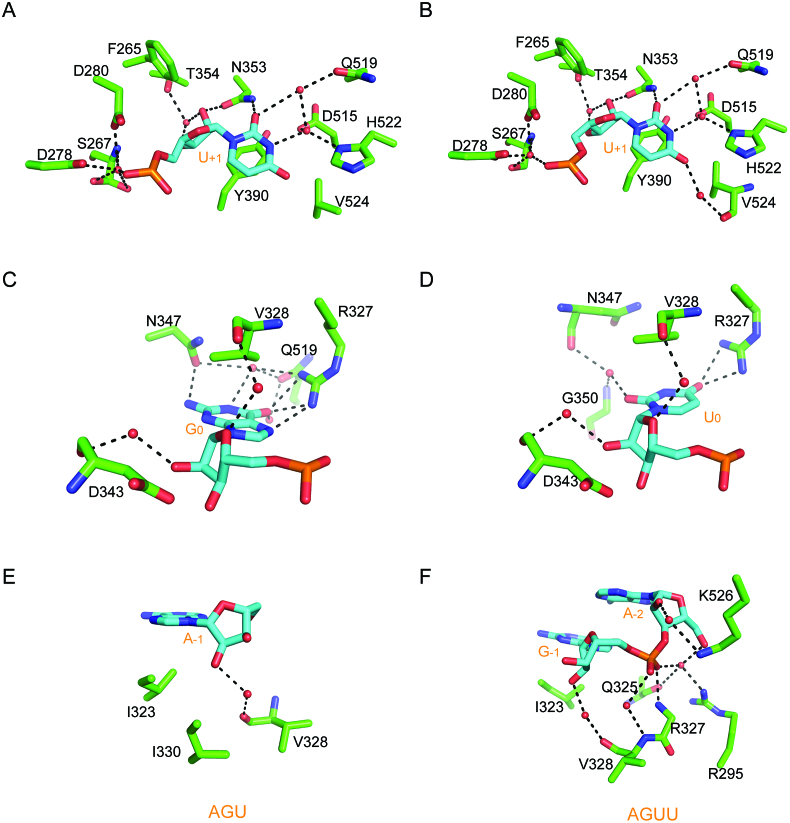
The detailed overview of Tailor's active sites. (**A, C**, **E**) Tailor-AGU interaction details at four different nucleotide-binding sites (–2, –1, 0 and +1 positions). (**B, D**, **F**) Tailor-AGUU interaction details at three different nucleotide-binding sites (+1, 0 and –1 positions). Nucleotides and surrounding residues are shown in cyan and green sticks, respectively. Hydrogen bonding interactions are all indicated as black dashed lines and water molecules are shown in red spheres.

As indicated by the structural analysis, the terminal Us in both Tailor-AGU and Tailor-AGUU adopt a very similar conformation at the +1 position (U_+1_) as their equivalent nucleotides in previously reported structures, interacting with the surrounding residues through extensive direct and water-mediated hydrogen bonds, as well as hydrophobic interactions (Figure 3A and B). More specifically, the O2 carbonyl group and 2′-hydroxyl group of U_+1_ make two direct hydrogen bonds with the side-chain group of N353. Furthermore, the side-chain of T354 contributes to the generation of water-mediated hydrogen bonds with 2′- and 3′-hydroxyl groups of U_+1_, while U_+1_ base forms hydrophobic stacking interaction against the aromatic side-chain of Y390 and an additional hydrophobic interaction with the main chain of V542, as well as a water-mediated hydrogen-bonding network with D515, Q519, and H522 from NRM loop. Complicated water-mediated hydrogen bonds are also observed between the phosphate moiety of U_+1_ and residues S267, D278 and D280. The minor difference between two complex structures at this active site is that V524 forms an extra water-mediated hydrogen bond in Tailor-AGUU. Among the residues involved into the interactions with U_+1_, Tailor N353, Y390 and H522 are highly conserved across the TUTases from different species (Supplementary Figure S1).

At the 0 nucleotide-binding position which is occupied by a guanine (G_0_) and a uridine (U_0_) in two complexes, respectively, protein-RNA recognition modes are similar but not identical (Figure 3C and D). In Tailor-AGU, the O6 carbonyl group and N7 imino proton of G_0_ form totally three hydrogen bonds with the guanidine groups of R327 from the loop connecting β3 and β4 strands while the NH2 group of G_0_ is involved in a hydrogen bond with the side-chain group of N347, ensuring this guanine is specifically recognized by Tailor. Other than these direct recognitions, G_0_ base also makes water-mediated hydrogen bonding interactions with the side-chain groups of N347 and Q519, and a hydrophobic interaction with the side-chain group of V328. In Tailor-AGUU, however, R327 only maintains two hydrogen bonds with O4 carbonyl group of U_0_ base whereas the direct hydrogen bond N347 side-chain participating in Tailor-AGU is missing. On the other hand, U_0_, using its base, forms water-mediated hydrogen bonds with the side-chain groups of N347 and G350 (not Q519) and a hydrophobic interaction with V328, similarly as G_0_ in Tailor-AGU. In addition, in both complex structures, U_0_ sugar ring makes two same water-mediated hydrogen bonds with the main chain of V328 and D343. The β3–β4 loop containing R327 contributes significantly to the recognition specificity for 3′ terminal guanine and uridine at the 0 position and is therefore termed as substrate-specificity loop (SSL) in this research (Figure 2A and Supplementary Figure S1).

5′A at the -1 position (A_-1_) in Tailor-AGU is less restricted by the interactions with the protein except the hydrophobic interactions with I323 and I330 and a water-mediated hydrogen bond between 2′-hydroxyl group and main chain atom of V328 (Figure 3E). In Tailor-AGUU, however, the phosphate group of G_-1_ forms a direct hydrogen bond with R327 and three more water-mediated hydrogen bonds with R295, Q325 and V328, besides those above-mentioned interactions for A_-1_ (Figure 3F). Most of these interacting residues are also the part of SSL which is highly conserved in different TUTases (Supplementary Figure S1). As the extra nucleotide in Tail-AGUU, A_-2_ makes no important interactions with Tailor but a water-mediated hydrogen bond between its 2′-hydroxyl group and K526 side-chain. Its conformational stability in this structure could be explained by the base stacking with the adjacent G_-1_.

### R327 and N347 contribute significantly to Tailor's recognition selectivity for 3′G

**Figure 4.**
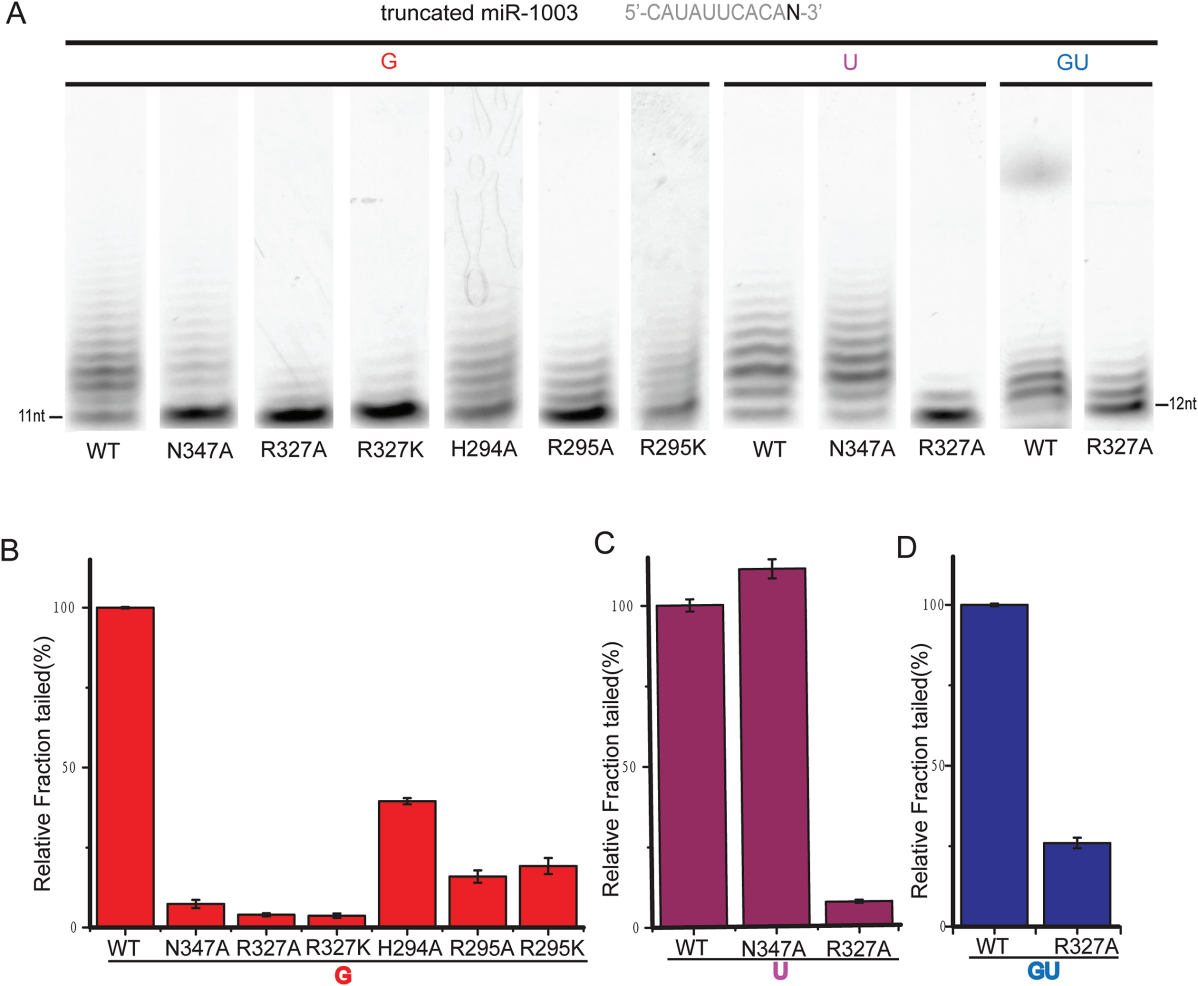
R327 and N347 are residues contributing significantly to Tailor's selectivity for 3′G. (**A**) *In-vitro* nucleotide transferase assays of truncated miR-1003 bearing 3′G, 3′U, or GU-3′ against wild-type Tailor-C and different mutants. Only the gel lanes containing the reactions stopped at 30-min time-point are shown for comparison, and the full gel results are shown in Supplementary Figure S5. (**B**–**D**) The comparison of tailing efficiency. The columns represent the tailed fractions of truncated miR-1003 bearing 3′G, 3′U, or GU-3′ in the assay. The fraction tailed by wild-type Tailor is normalized to 100%. Each assay was repeated for three times. Each of three replicated was independently analyzed and mean values and standard deviations are depicted.

As indicated above, R327 and N347 are key residues in forming direct hydrogen bonds with G_0_. To verify whether these specific interactions are responsible for the intrinsic preference of Tailor for RNA substrate with 3′ terminal G, two alanine mutants (R327A and N347A) were generated for in-vitro nucleotide transferase assay. Our results summarized in Figure 4 and Supplementary Figure S5 indicated, for the RNA substrate ending in 3′G, the uridylation efficiencies of both mutants reduced dramatically when compared with that of wild-type Tailor. Based on the analysis of the data collected at the 30-min time-point, the enzymatic activities of R327A and N347A drop by ∼96% and ∼93%, respectively. To further investigate whether R327 has to be strictly conserved, we also generated a lysine mutant of R327 (R327K) for enzymatic activity assay. Surprisingly, our result that R327K also lose ∼96% enzymatic activity compared to the wild-type Tailor further indicated arginine turns out to be the amino acid required for this residue position to ensure Tailor's selectivity for RNA substrate. In addition, EMSA was also employed to check the mutants listed above and our results showed that, compared with wild-type Tailor-C, all mutants exhibit evident reductions in the binding of Tailor with truncated miR-1003 (Supplementary Figure S6). Collectively, our evidences showed that R327 and N347 are crucial residues contributing to Tailor's intrinsic preference for RNA substrate ending in 3′G.

Considering R327 also contributes to the direct hydrogen interactions with U_0_ in the complex structure of Tailor-AGUU, in-vitro nucleotide transferase assay was again employed to investigate the effects of Tailor mutants on its enzymatic activity against the RNA substrate ending in 3′U (Figure 4A, C and Supplementary Figure S5). Our results showed that R327A maintains a ∼93% reduction in uridylation efficiency whereas N347A causes no significant effect on it (∼11% increase), in line with our structure information. Moreover, simple replacement of G_0_ and U_0_ bases with those of A and C in our complex structures show no possibilities for them to form direct hydrogen bonds with R327 and N347 if A and C follow the similar conformations of G_0_ and U_0_ (Supplementary Figure S7), further supporting our experimental results. In addition, we determined a crystal structure of Tailor-C in complex with an oligoU stretch (5′-UUUU-3′), in which three uridines at the 3′ side, except the 5′U, all exhibit clear electron densities and occupy +1, 0, and –1 nucleotide-binding positions (Supplementary Figure S4C). The similar recognition preferences of Tailor exhibiting for U_0_ in both structures of Tailor-AGUU and Tailor-U4 are believed to favor the extension of polyuridylation chain (Figure 3D and Supplementary Figure S8). Therefore, R327 may undertake a second task in the tailing process in addition to cooperate with N347 to provide a recognition platform for 3′G. To testify this hypothesis, R327A mutant was applied to the enzymatic activity assay against a RNA substrate ending in GU-3′. Our result showed that R327A also results in an evident reduction in tailing efficiency towards wild-type Tailor (Figure 4A, D and Supplementary Figure S5), suggesting a conclusion that Tailor R327 plays two similar but not identical roles during the 3′ tailing process of RNA: providing interaction specificity in the initial recognition of 3′G and anchoring uridine for the subsequent extension of polyuridylation chain.

### 3′G preference of Tailor requires the co-participation of R327 and N347

**Figure 5.**
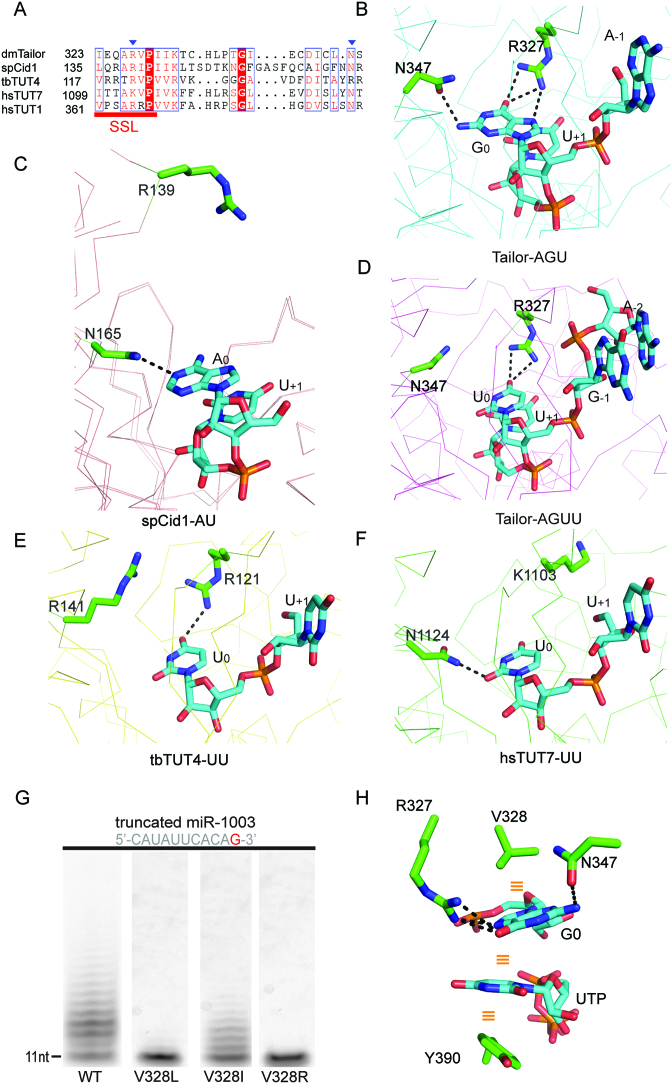
Sequence and structure conservations of R327 and N347. (**A**) Sequence alignment of Tailor with other TUTases, including *sp*Cid1, *tb*TUT4, *hs*TUT7 and *hs*TUT1. Numbers before the sequence indicate the residue number of the first amino acid in the sequence shown. R327 and N347 are highlighted by blue reverse triangles. (**B**–**F**) Structural comparisons of R327 and N347 in Tailor-AGU and Tailor-AGUU with their equivalent residues in complex structures of *sp*Cid1-AU (PDB ID 4NKU), *tb*TUT4-UU (PDB ID 5KAL), and *hs*TUT7-UU (PDB ID 5W0N). The hydrogen bonds between these residues and the nucleotides at the 0 position are indicated as black dashed lines. (**G**) *In-vitro* nucleotide transferase assays of truncated miR-1003 bearing 3′G against wild-type Tailor-C and three mutants of V328 (V328I, V328L, and V328R). (**H**) The protein-RNA stacking network in the active site of Tailor-AGU. The UTP from Tailor-UTP is superimposed with U_+1_ to show the conformations of uridine base in these two structures are exactly same. The stacking interactions are all indicated as orange lines.

Sequence alignment reveals that R327 and N347 contributing to the Tailor's preference for 3′ terminal guanine are quite conserved in TUTases from other species, including *Schizosaccharomyces pombe, Trypanosoma brucei*, and *Homo sapiens* (Figure 5A and Supplementary Figure S1). This finding is somewhat surprising since no 3′ end preference of TUTase for RNA substrate has been so far reported for other species other than flies. To further learn the structural conservation, we superimposed our complex structure of Tailor with all previously reported structures of other TUTases complexed with short RNA substrates (*sp*Cid1-AU, *tb*TUT4-UU and *hs*TUT7-UU) (Figure 5B–F and Supplementary Figure S9). In Tailor-*sp*Cid1 comparison, Cid1 R139 exhibits a largely different conformation from Tailor R327, making no contacts with the adenine at the 0 position. Cid1 N165, on the other hand, maintains a similar conformation as Tailor N347 and forms direct hydrogen bond with the adenine (Figure 5C). In *tb*TUT4-UU, the equivalent residue of Tailor N347 is an arginine (R141) which possesses a side-chain with different orientation from that of N347 and makes no hydrogen bonding interaction with U_0_ whereas *tb*TUT4 R121 plays a same role as Tailor R327 in anchoring U_0_ through a hydrogen bond (Figure 5E). In *hs*TUT7-UU, however, N1124 maintains a similar hydrogen bond with U_0_ as Tailor N347 while K1103 (equivalent to Tailor R327) forms no interaction with it (Figure 5F). Based on above observations, we concluded that the 3′G preference of TUTase for RNA substrate probably needs two criteria: the strict residue conservation at the positions of R327 and N347 of Tailor, and the cooperative participation from these two residues.

As the immediately adjacent residue to R327, V328 is less conserved compared with R327 and N347, and observed to form hydrophobic stacking with 3′ nucleotide base in our complex structures. The tailing assay of its reverse mutations (V328L, V328R and V328I) showed that both V328L and V328R entirely abolished Tailor's catalytic activity for truncated miR-1003 bearing 3′G while V328I also exhibited a obvious reduction in uridylation efficiency (Figure 5G), implicating a significant contribution of V328 to Tailor's enzymatic function through establishing a sequential side-chain-to-base stacking network (V328-3′G-UTP-Y390) in the active site of Tailor (Figure 5H). EMSA was further employed to show that all three V328 mutants barely affected Tailor's binding with RNA substrate (Supplementary Figure S6). We proposed that the 3′G base within this stacking network is further stabilized by collaborative hydrogen bonding interactions from R327 and N347 in an optimal conformation for efficient uridylation reaction.

### Conformational stability of the exit of RNA-binding groove regulates Tailor's activity

**Figure 6.**
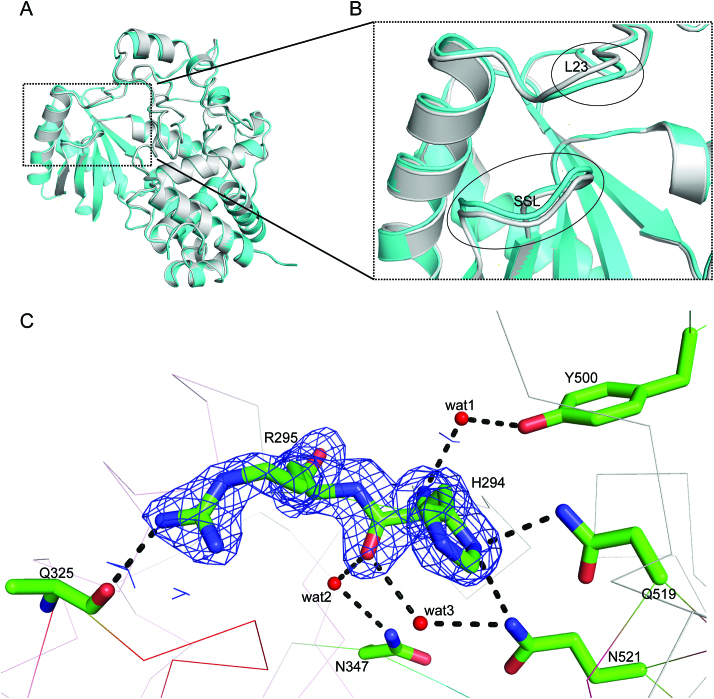
Conformational stability of the exit of catalytic groove changes upon RNA binding. (**A**) The superimposition of crystal structures of apo-form Tailor (gray) and Tailor-AGUU (cyan). (**B**) A close-up of SSL and h23 loops in two structures. Both loops shift towards each other upon RNA binding. (**C**) H294/R295-mediated hydrogen-bonding network in Tailor-AGUU. All residues are shown in sticks as well as the electron density map with 2Fo-Fc calculated at 1.0σ for H294 and R295. Hydrogen bonding interactions are all indicated as black dashed lines and water molecules are shown in red spheres.

Although the overall structures of Tailor in apo and RNA-bound forms are highly similar, careful comparison did reveal a structural perturbation, upon the RNA binding, occurring on a region next to the exit of RNA-binding groove (Figure 6A and B), which is mainly constituted by H294 and R295 from the loop connecting h2 and h3 (L23), R327 from SSL loop, as well as N521 and K525 from NRM loop (Supplementary Figure S1). Except R327 which participates into the hydrogen bond with the nucleotides at the 0 position in two complex structures, other positively charged residues make no direct interactions with RNA. Specifically, in RNA-bound structures, the portion of L23 loop (H294 and R295) and SSL loop slightly shifted towards each other upon the RNA binding, forming a direct hydrogen bond between the side-chain of R295 from SSL and the main chain atom of Q325 from L23 (Figure 6C), which is not observable in the apo-form structure of Tailor. In addition, the side-chain of H294 forms two direct hydrogen bonding interactions with Q519 and N521 while H294 also forms three water-mediated hydrogen bonds with N347, Y500 and N521 using its main chain atoms (Figure 6C), in both complex structures. All these H294-related hydrogen bonds were not observed in the apo-form structure of Tailor, reflecting a higher degree of rigidity for this region in the RNA-bound structures, which is consistent with its B-factor profiles in three models (Supplementary Figure S10). To evaluate whether this H294/R295-mediated hydrogen-bonding network is important for Tailor to facilitate its function as RNA TUTase, we generated their mutants (H294A, R295A, and R295K) for in-vitro nucleotide transferase assay. Our results showed that the enzymatic activities of R295A and R295K reduced significantly by ∼84% and ∼80% while noticeable effect (∼61% reduction) is also observed for H294A (Figure 4A, B and Supplementary Figure S5), suggesting the important roles of R295 and H294 in Tailor's function. We proposed that R295, probably with the help of H294, stabilizes the conformation of the exit of RNA-binding groove in Tailor through the formation of a local hydrogen-bonding network, generating a structural environment beneficial to RNA binding. This speculation was supported by our EMSA results of R295 and H294 mutants (Supplementary Figure S6).

## DISCUSSION

The preference for RNA substrate with 3′ terminal guanine is a unique feature recently identified in *Drosophila* TUTase Tailor (40,41). Although the enzymatic domain of TUTases are highly conserved across different species and its structure-and-function relationship has been well illustrated by a series of investigations (16,17,19–22,56), the structural basis for this novel 3′G preference remains mysterious to the field. Here, using crystallography, we solved multiple structures of Tailor enzymatic domain and its complexes with 5′-AGU-3′, 5′-AGUU-3′, and 5′-UUUU-3′ RNA stretches, which could be put together to describe an intact process of Tailor-mediated oligouridyation occurring at the 3′G of RNA substrate. Our structural information as well as the results of *in-vitro* nucleotide transferase assay indicated that R327 and N347 are residues contributing significantly to Tailor's 3′G preference probably through stabilizing a 3′G-participated stacking network (V328-3′G-UTP-Y390) in the active site of Tailor and R327 plays an extra role in facilitating the efficient extension of polyuridylation chain. Although R327 and N347 are highly conserved in the TUTases across different species, our analysis and experimental evidence suggested that both strict sequence and structure conservation are necessary for these two residues to maintain the 3′G preference in a TUTase.

**Figure 7.**
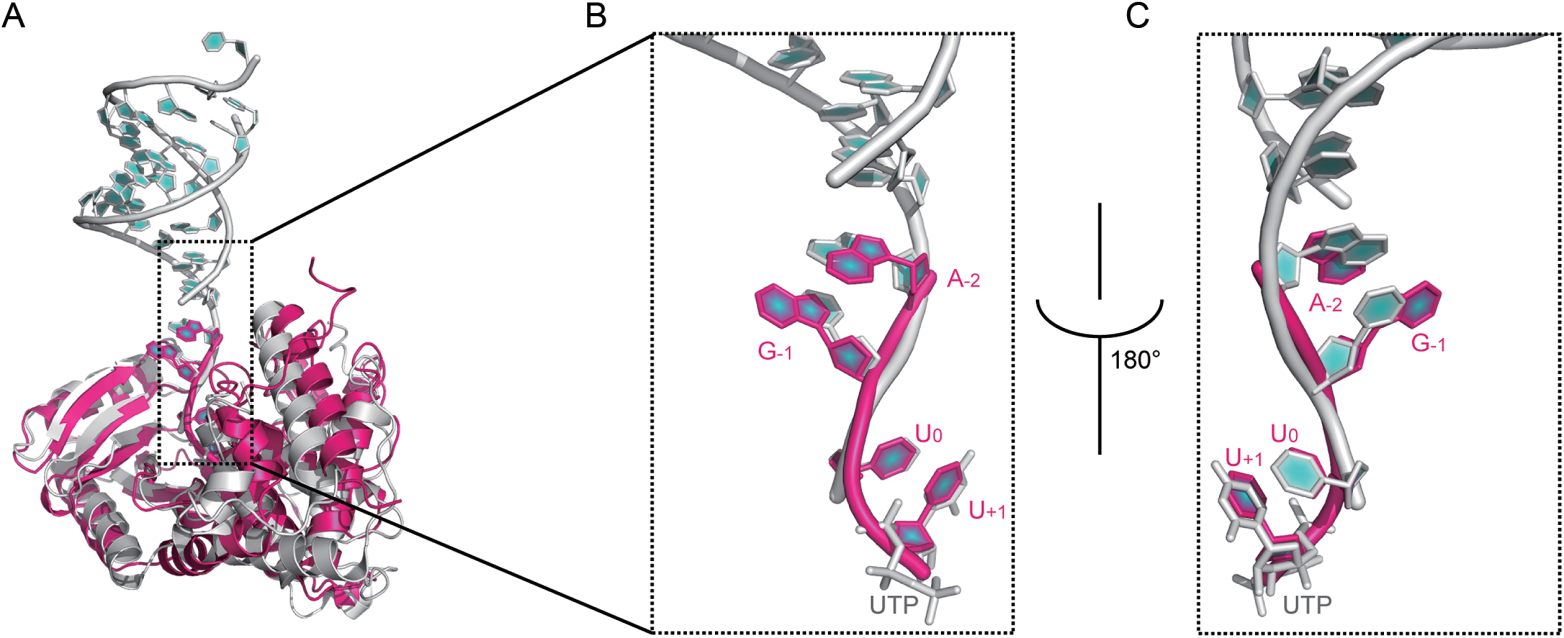
Structure comparison of Tailor-AGUU and *hs*TUT7-dsRNA. (**A**) Overall comparison of two complex structures by superimposing the enzymatic domains of Tailor (red) and *hs*TUT7 (gray). (**B, C**) The close-ups of the structural comparison of 5′-AGUU-3′ (red) with dsRNA and UTP (gray). The portion of the single strand complementary to the nucleotides aligned with G_-1_ and A_-2_ of 5′-AGUU-3′ in ds-RNA was removed for a clearer effect.

One remaining concern regarding the validity of our complex structure is that authentic mirtron miR-1003 is a 56-nt long RNA hairpin ending in a 2-nt 3′ overhang (AG-3′) while only single-stranded 5′-AGU-3′ or 5′-AGUU-3′ is used in our complex structures. Through a structural comparison of Tailor-AGUU and recently reported *hs*TUT7-dsRNA complex (PDB ID 5W0O) in which a double-stranded RNA is designed to represent the duplex stem of group II pre-let-7 and contains a 1-nt 3′U overhang (56), we find that the conformations of AGUU-3′ in our complex structure can be almost perfectly aligned with those of GCU-3′ from one of the single strands of dsRNA plus UTP in *hs*TUT7-dsRNA structure (Figure 7) despite the GC is part of RNA duplex while the AG in our structure is single-stranded. This structural similarity suggests that, during the process that Tailor catalyzes the addition of second uridine onto AGU-3′ of RNA substrate, the adenine and guanine will form a purine–purine base stacking to mimic the RNA duplex and endow the 3′-end of RNA substrate with higher rigidity. Therefore, it is very likely that our Tailor-AGUU structure reflects the real protein-RNA recognition between Tailor and its mirtron RNA substrates

There remain open questions regarding the biological significances of biogenesis pathway of mirtron in which Tailor plays a crucial role. For instance, Tailor mutant flies have reduced fertility and the underlying mechanism is still unknown (42,48). The mutants of R327 and/or N347 we generated in this research have a useful value in the function studies of Tailor-mediated uridylation of mirtron in flies when these mutants, especially R327K, could be applied to precisely abolish Tailor's 3′G preference without sacrificing its fundamental TUTase activity.

## DATA AVAILABILITY

Coordinates and structure factors for apo-form Tailor and Tailor-RNA complexes (Tailor-AGU, Tailor-AGUU, Tailor-U4 and Tailor-UTP) have been deposited in the Protein Data Bank under accession code 5Z4C, 5Z4A, 5Z4D, 5Z4J and 5Z4M.

## Supplementary Material

Supplementary Data
