# Supplementary material for: Structural insights into a unique preference for 3′ terminal guanine of mirtron in Drosophila TUTase tailor
Source: Nucleic Acids Res. 2018 Nov 8;47(1):495–508. doi: 10.1093/nar/gky1116 (PMC6326804; doi:10.1093/nar/gky1116)
Supplement: Supplementary Data [file gky1116_supplemental_files.pdf]

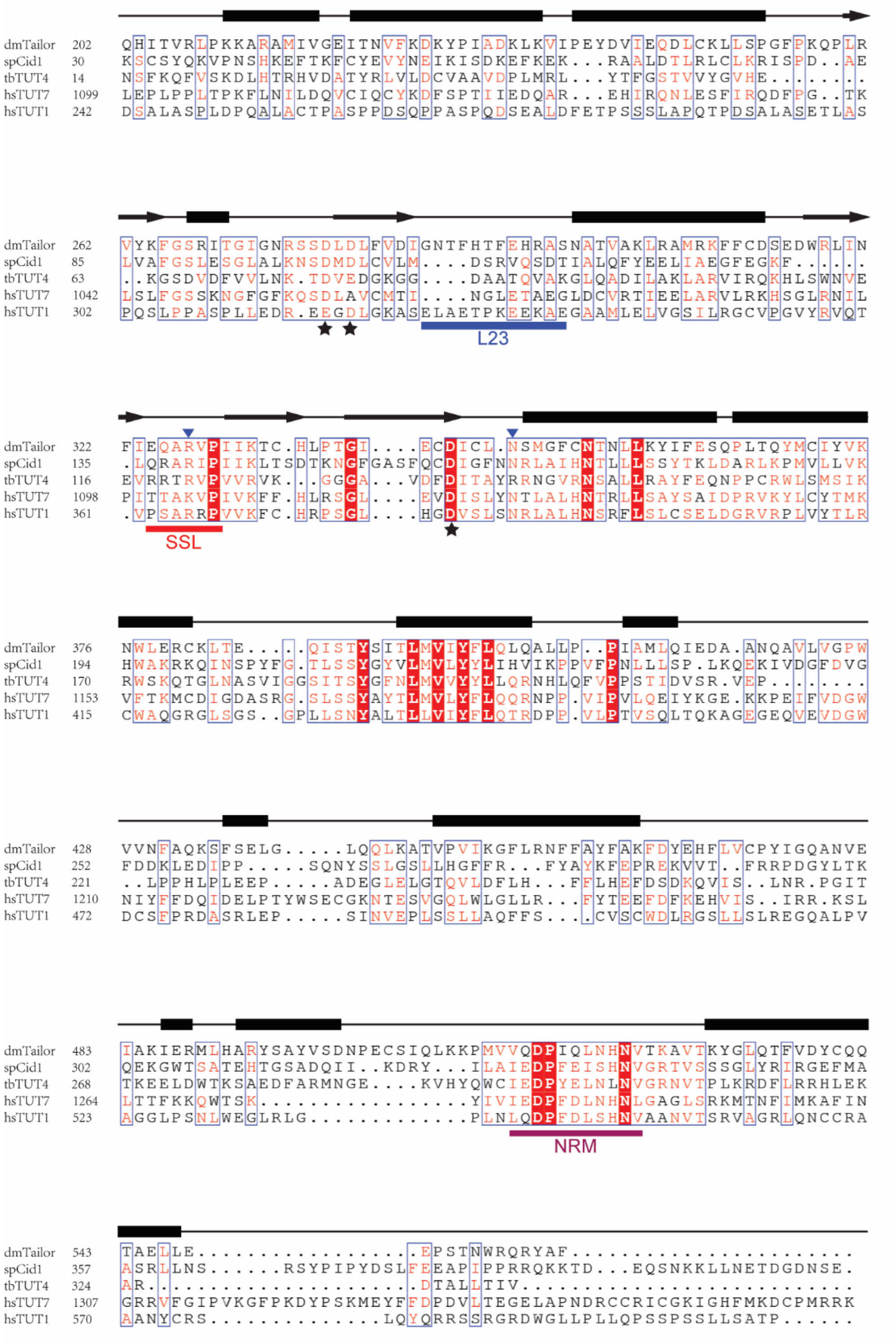

**Figure S1**. Sequence alignment of Tailor with other TUTases, including *sp*Cid1, *tb*TUT4, *hs*TUT7, and *hs*TUT1. Numbers before the sequence indicate the residue number of the first amino acid in the sequence shown. Secondary structure elements are displayed at the top of the sequence. Three important loops, including NRM, SSL, and L23, are underlined in purple, red, and blue, respectively. Key residues (R327 and

N347) we identified for 3′G preference of Tailor in this research are highlighted by blue reverse triangles, and three conserved catalytic residues (Asp278, Asp280, and Asp343) are marked by black asterisks.

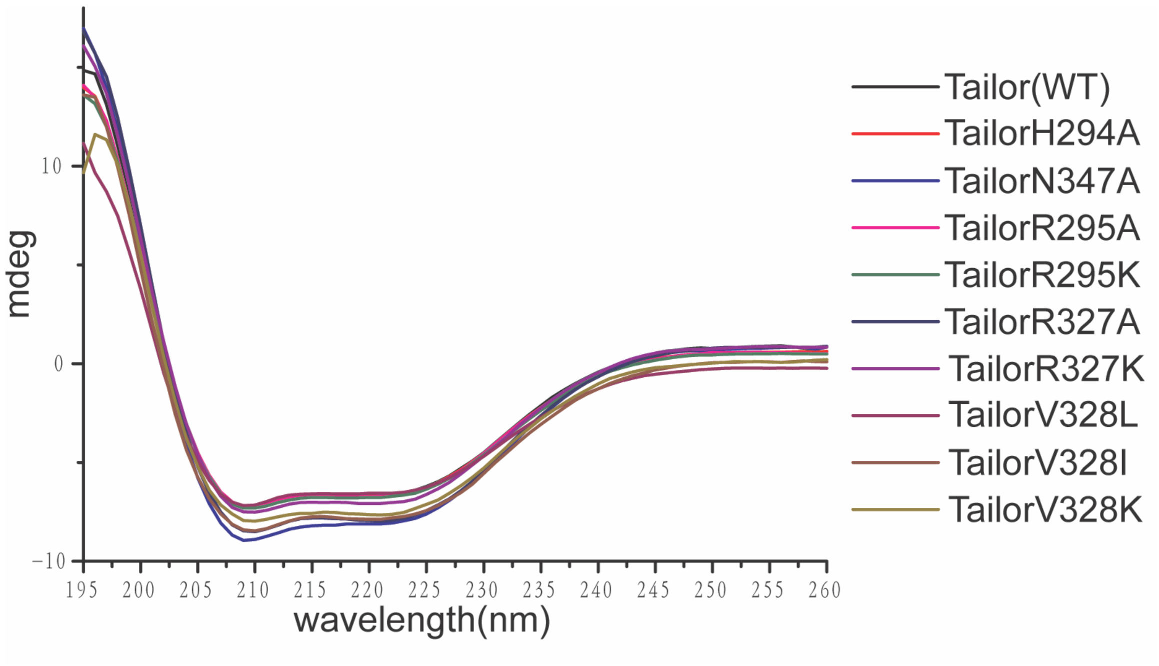


**Figure S2**. CD spectra of wild-type Tailor-C and its mutants.

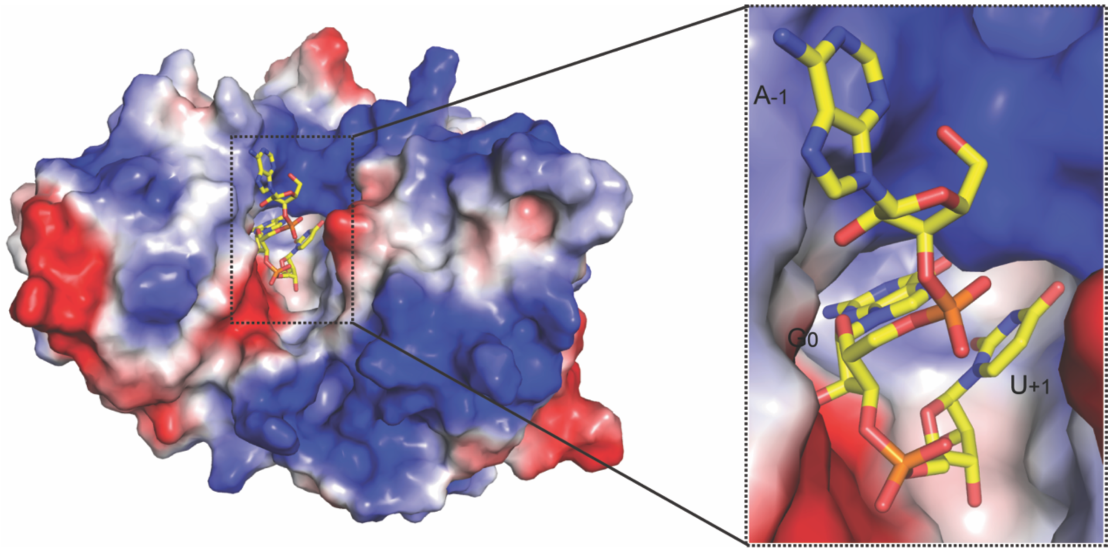


**Figure S3**. The overview of 5′-AGU-3′ lying along the catalytic groove of Tailor. Tailor-C is shown in its electrostatic surface potential, and RNA stretches are shown in stick model. (*Inset*) A close-up of the engagement of 5′-AGU-3′ into the catalytic groove.

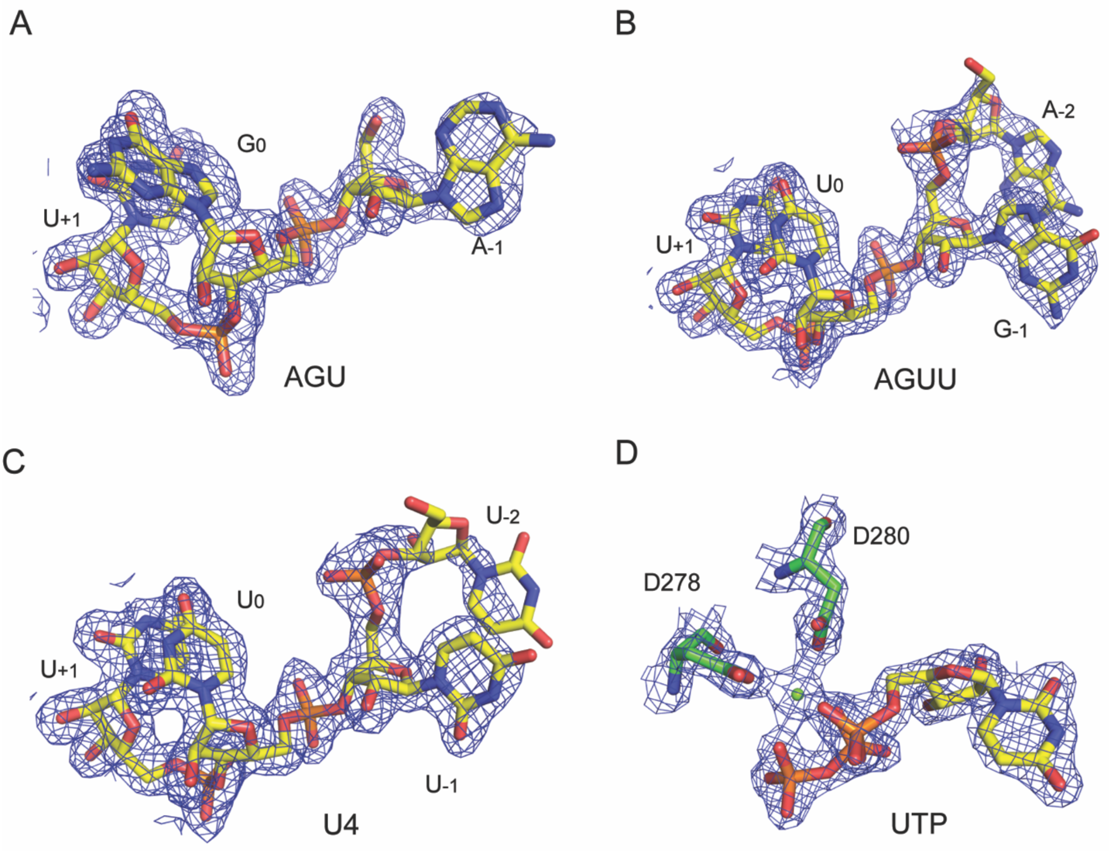


**Figure S4**. (**A-D**) The nucleotides in four complex structures of Tailor-C are shown in sticks as well as the the electron density map with 2Fo-Fc calculated at 1.0σ for 5′-AGU-3′, 5′-AGUU-3′, U4 and 2.0σ for UTP. A magnesium ion in D is represented in a green sphere.

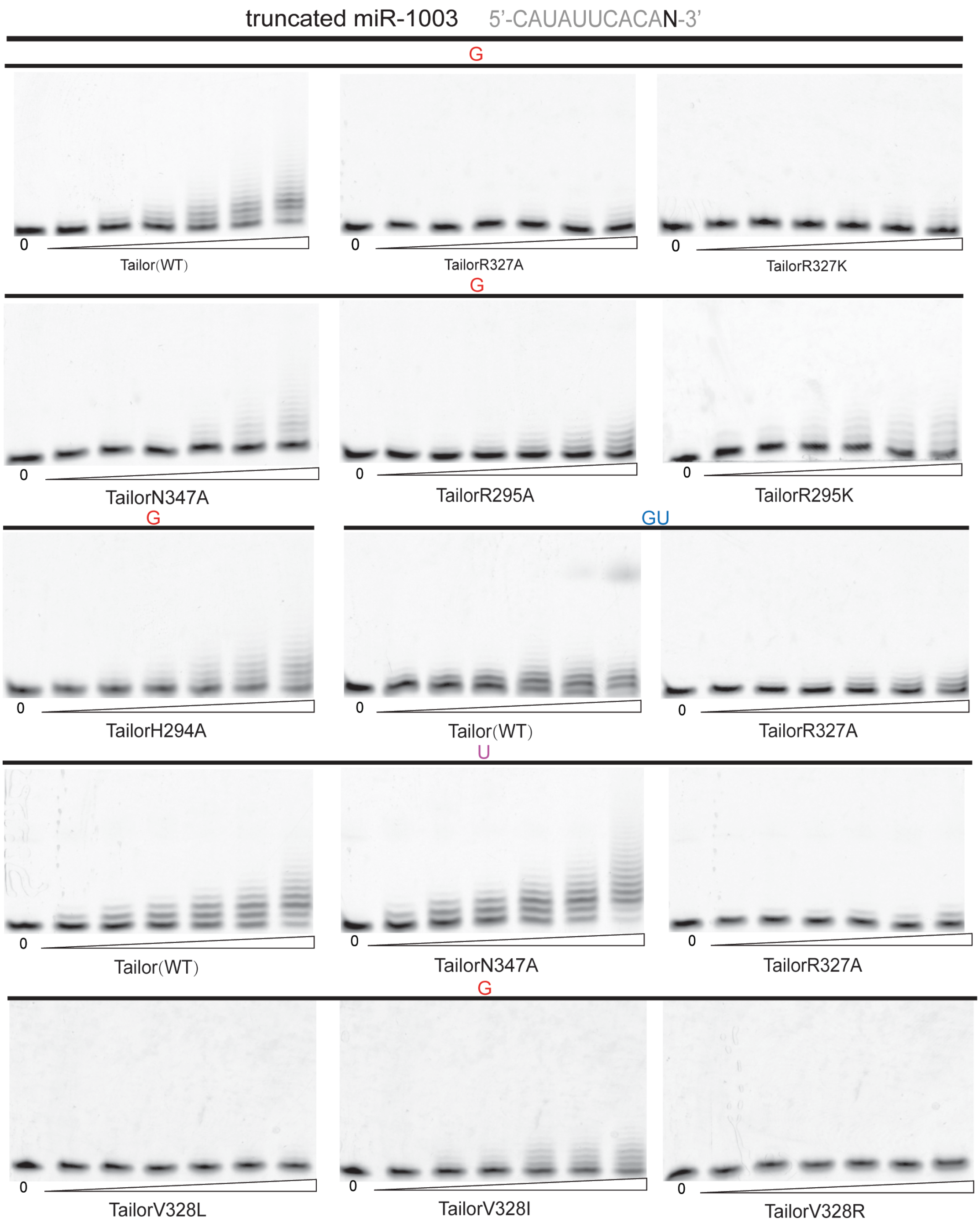


**Figure S5**. Full results of gel-based nucleotide transferase assays of truncated miR-1003 bearing 3′G, 3′U, or GU-3′ against wild-type Tailor-C and different mutants.

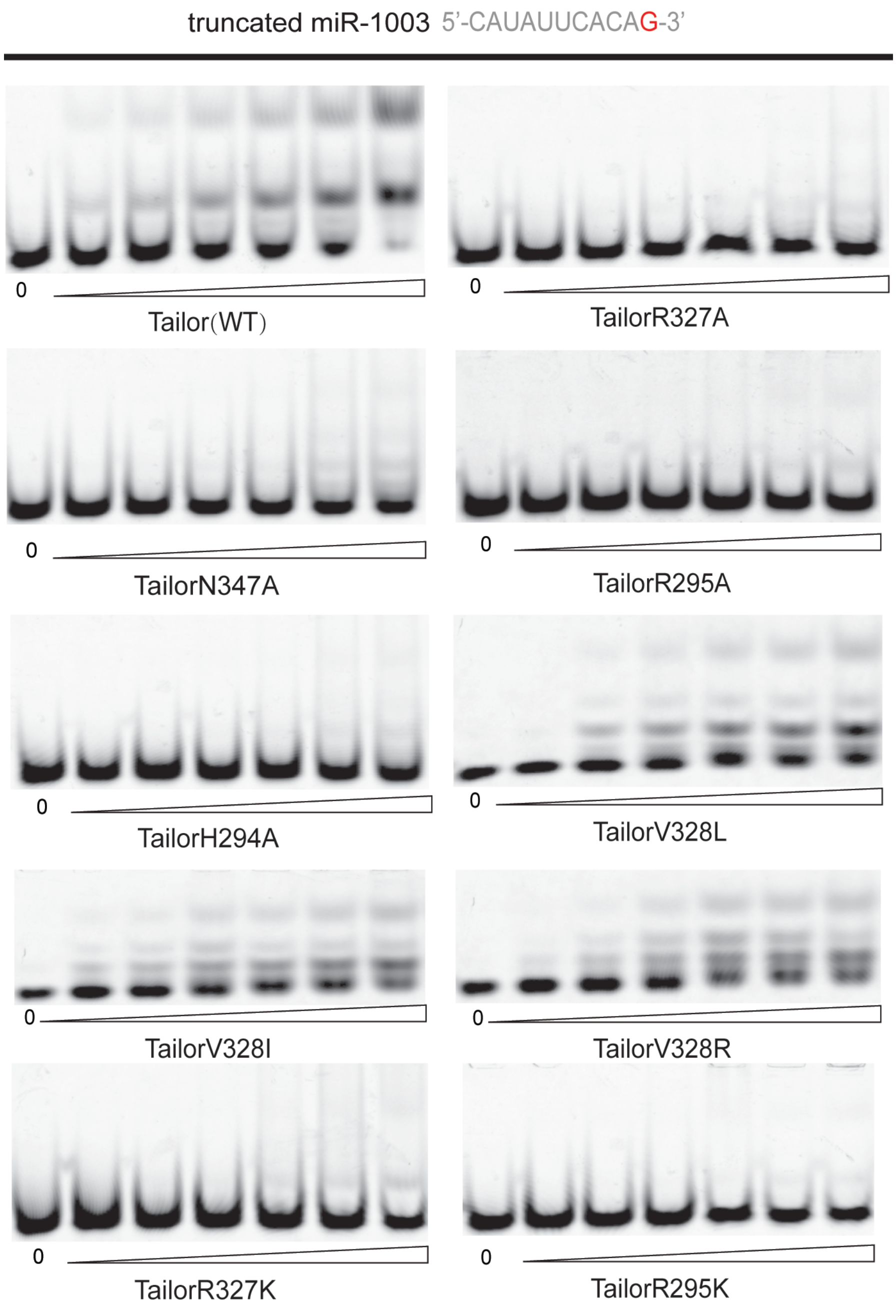


**Figure S6**. EMSA results of the binding of wild-type Tailor-C and its mutants with truncated miR-1003.

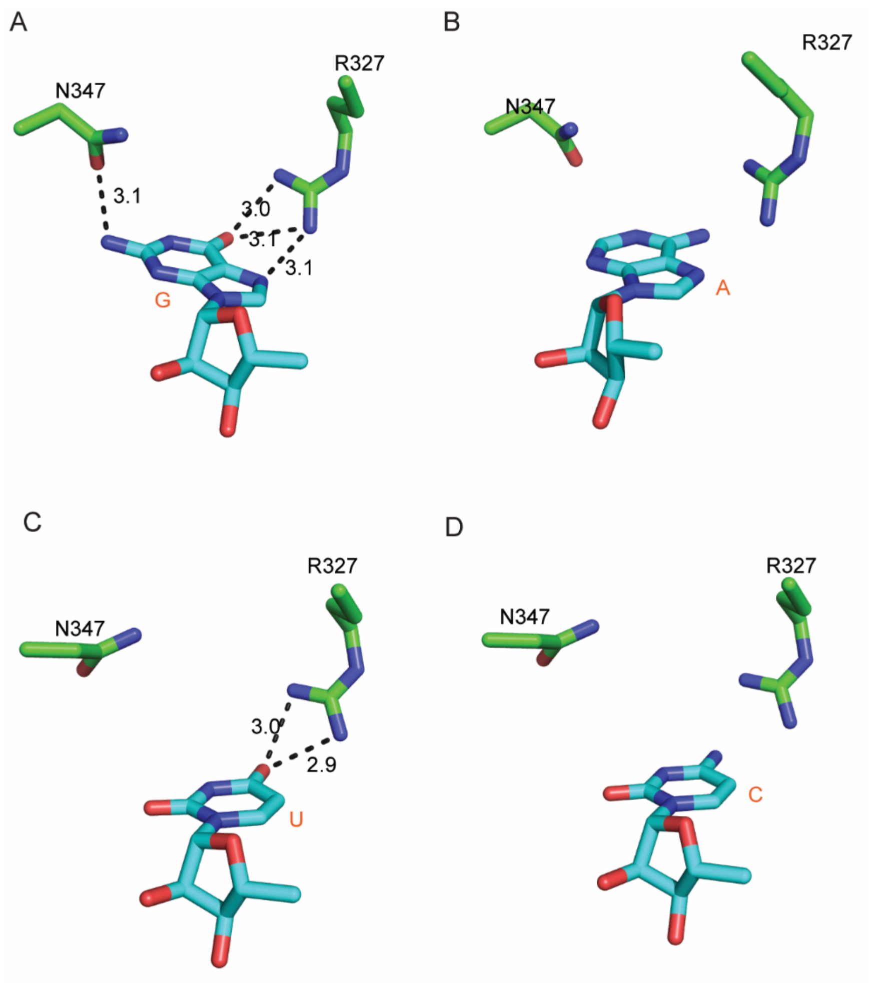


**Figure S7**. Simple replacement of $G_0$ (**A**) and $U_0$ (**C**) bases with those of adenine (**B**) and cytosine (**D**) in our complex structures show no possibilities for them to form direct hydrogen bonds with R327 and N347 if adenine and cytosine follow the similar conformations of $G_0$ and $U_0$.

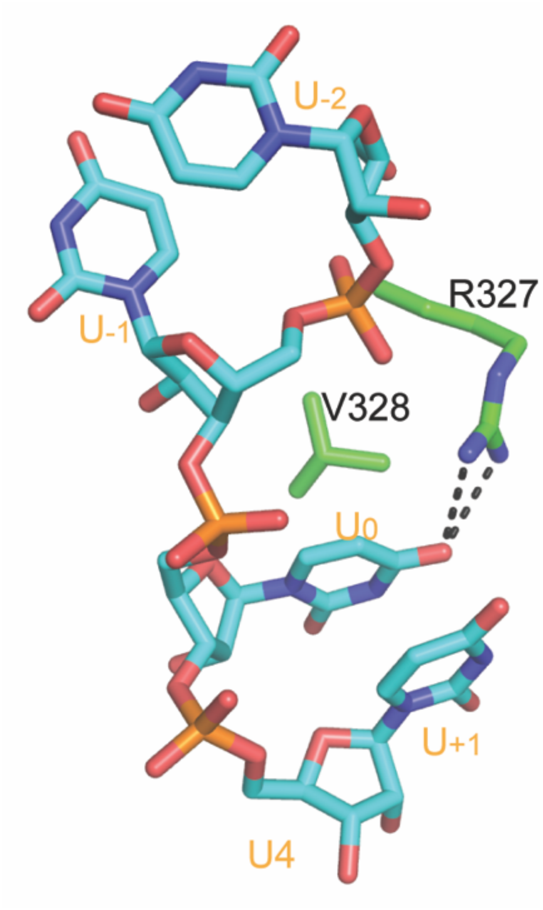


**Figure S8**. Interaction details between U4 (5′-UUUU-3′) and R327 of Tailor. Hydrogen bonding interactions are all indicated as black dashed lines.

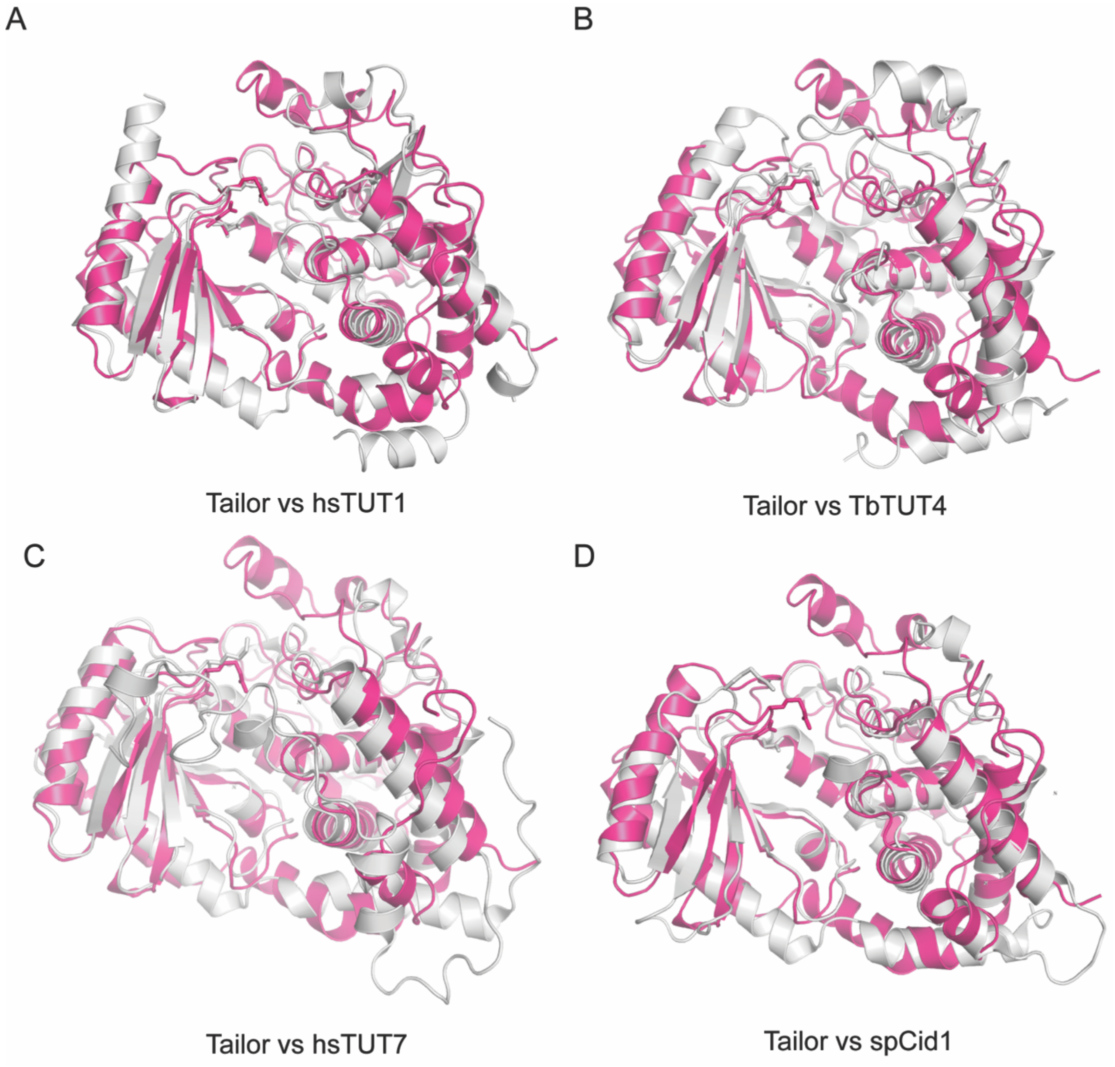


**Figure S9**. The structural superposition of Tailor-AGUU (red) and other TUTases (gray). The RMSD values of Cα superposition are 2.5Å for apo-form *hs*TUT1 (PDB ID 5WU1), 2.6Å for *tb*TUT4-UU (PDB ID 5KAL), 2.8Å for *hs*TUT7-UU (PDB ID 5W0N), 2.8Å for *sp*Cid1-AU (PDB ID 4NKU).

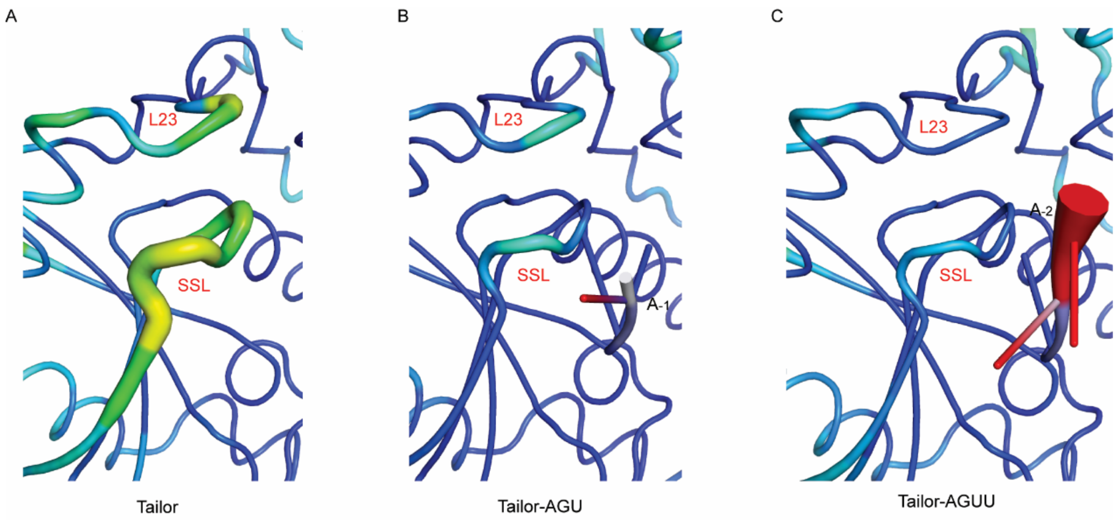


**Figure S10**. Comparison of B-factor profiles of SSL and L23 loops in apo-form Tailor (**A**) and its complexes with 5′-AGU-3′ (**B**) and 5′-AGUU-3′ (**C**). B-factor analysis suggests higher degree of rigidities are related to these two loops in the complex structures than apo-form structure. The thickness of the protein backbone is proportional to the B-factors of Cα atoms.
